# Reliability of the single leg stance test for safe removal of external fixator after tibial bone lengthening

**DOI:** 10.1038/s41598-025-14584-x

**Published:** 2025-08-17

**Authors:** Mahmoud El-Rosasy, Osama Elgebaly, Amr Elrosasy, Abdullah Khaled

**Affiliations:** 1 Department of Orthopedic Surgery, Faculty of Medicine, Tanta University, Tanta, Egypt; 2 Cairo university, Giza, Egypt

**Keywords:** Single leg stance, Tibial lengthening, Regenerate, Ilizarov, Bone transport, Fracture repair, Trauma

## Abstract

The timing of fixator removal in distraction osteogenesis is an important decision. Bipedal weight bearing may not be an accurate estimate of body weight distribution on both limbs. The purpose of this study was to test the reliability of the Single leg stance test (SLST) as an indicator of regenerate bone maturation and safe removal of the external fixator. Patients who underwent Ilizarov limb reconstruction for tibial bone lengthening were classified into two groups. The decision to remove the external fixator was based on radiological analysis and Bipedal walking in group A versus SLST in Group B. to be included in the study, the patient should be able to communicate, perform the test on the healthy limb, have no neuromuscular, visual or vestibular disorders, can do bipedal weight bearing unsupported and plain radiographs show at least three intact cortices. The data was collected retrospectively from 2012 to 2015 in group A and prospectively from 2016 to 2022 in group B. Interpretation of the test was done by two experienced surgeons in limb reconstruction and a junior orthopedic surgeon. A total of 50 patients and 52 patients were included in group A and B respectively. The inter-observer reliability of the three observers was almost perfect. Refracture after external fixator removal occurred in five cases in group A (10%) and one case in group B (1.9%). The SLST was able to predict regenerate maturation in 98% of cases in our series.The SLST is a simple, reproducible, and reliable clinical test that can be used to evaluate the maturation of the regenerate.

## Introduction

Distraction osteogenesis has been used extensively for limb reconstruction. Although distraction osteogenesis has solved limb length discrepancies, many complications are inevitable from long duration of external fixation^1,2^. The timing of fixator removal in distraction osteogenesis is an important decision because premature removal of the fixator can result in gradual bending or fracture of the regenerate^3,4^. Decisions regarding external fixator removal and weight bearing depend on the amount of callus seen in the distraction gap on radiographs. The presence of three of four continuous cortices on antero-posterior and lateral radiographs has been considered as an indication of consolidation of the regenerate bone^5,6^.

Determining when it is safe to remove the fixator can be difficult. Most surgeons used radiographs and clinical evaluation to determine timing of fixator removal. The decision to remove an external fixation device based on radiographic assessment alone resulted in intra-observer and inter-observer variability moderately above chance^7,8^.

Several attempts are being made constantly to establish objective guidelines for early and safe removal of a fixator using a sensitive and quantitative measurement technique. Various quantitative methods such as bone mineral density (BMD) assessment, ultrasound, Q-computed tomography, Dual-energy X-ray absorptiometry (DEXA) and pixel value ratio (PVR). Despite sensitive, these measures are expensive, and none has acquired gold standard status^3,5,9,10^.

Iobst et al. had surveyed an international group of external fixation surgeons to determine their current practice patterns surrounding external fixator removal. The top 5 responses for determining when it is safe to remove a fixator were full weight bearing (75%), 3 cortices (71%), no pain (55%), after dynamization (55%), and duration of time (30%)^11^.

Bipedal weight bearing may not be an accurate estimate of body weight distribution on both limbs because pain and weakness of either limbs would alter the stresses imposed on the treated limb.

The single-leg stance test (SLST), also known as the unipedal stance test, is often used to assess static postural and balance control^12^. In our department, the SLST has been used, in addition to radiographs since several years as an indicator of bone consolidation and safe removal of the external fixator^13^.

The purpose of this study was to test the reliability of the SLST as an indicator of regenerate bone maturation and the utility of the test to supplement radiography in evaluating bone healing and in determining when the regenerate stiffness is sufficient for safe removal of the external fixator.

### Patient and methods

This was a combined retrospective and prospective cohort study which included patients who underwent Ilizarov limb reconstruction for tibial bone lengthening in a specialized center for limb lengthening and reconstruction. All participants provided written informed consent. The work was conducted in accordance with the ethical principles of the declaration of Helsinki. Ethical approval was obtained from faculty of medicine- Tanta university ethical committee with approval code (36264PR514). The study follows STARD guidelines for testing the reliability of SLST for prediction of safe removal of external fixator after tibial bone lengthening. Both bone transport to bridge bone defect and leg lengthening were the indications for surgery in the studied cases. The patients were classified into two groups: in (group A); the decision to remove the external fixator was based on combined radiological analysis and clinical evaluation (the ability for bipedal walking without using support or assisting devices) the data of this group was collected retrospectively from the patients records during the period from 2012 to 2015. The patients who didn’t complete the follow up in our institution for at least one year after fixator removal are excluded from the study. In 2016 the single leg stance test (SLST) was utilized in our center as an added test for clinical evaluation of the cases who underwent Ilizarov limb reconstruction before removal of the external fixator. To test for the reliability of the single leg stance test for safe removal of the external fixator after tibial bone lengthening, the data from Group B was prospectively collected during the period from 2016 to 2022. The decision of removal of the external fixator in (group B) was based on radiological evaluation of plain x rays ( for regenerate: to be homogenous, with adequate caliber, no interruptions with at least intact three cortices and for docking site presence of at least intact three cortices)and clinical evaluation using the SLST.

*Test requirements* to be included in the study the patient should fulfill the following criteria: (1) ability to communicate; (2) can perform the test on the healthy limb; (3) no neuromuscular disorders; (4) no vestibular or visual disturbances; (5) can do bipedal weight bearing unsupported; and (6) plain radiographs show at least intact three cortices.

*Test description* the patient stands bare footed and is requested to stand on the healthy limb and lift the treated limb and stay steady and unsupported for 30 s. If managed to do the first part of the test, then the patient is asked to repeat the same test on the treated limb. For the test to be considered as positive the patient should be able to stand on the treated limb for a minimum of 30 s with other limb lifted off the ground.

**Fig. 1:**
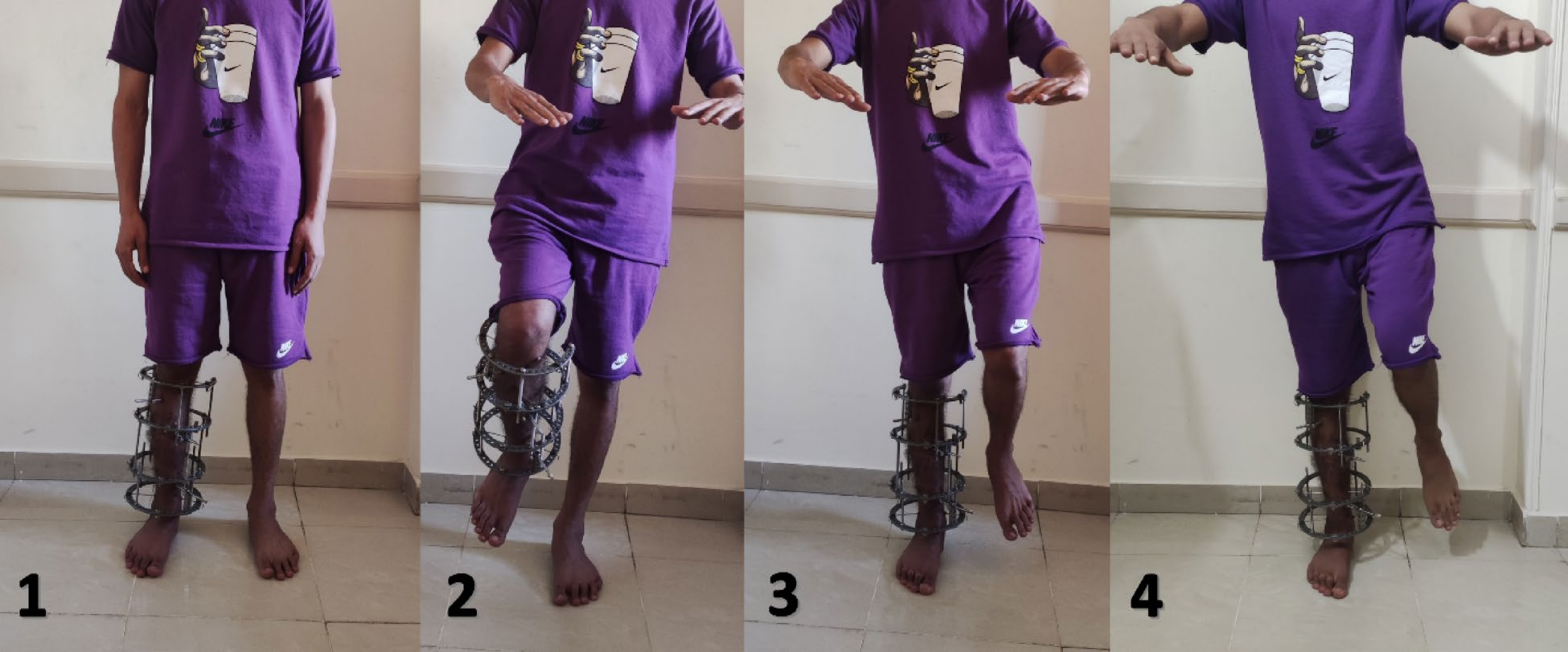
Demonstration of SLST Steps. 1: Bipedal full weight bearing unsupported. 2: SLST on the healthy limb. 3: SLST on the treated limb while fixator is connected. 4: SLST on the treated limb while the frame is destabilized.

*Test design* the sequence of test performance follows certain steps. (Fig. 1)

*Step 1* ensure bipedal full weight bearing unsupported.

*Step 2* do SLST on the healthy limb.

*Step 3* do SLST on the treated limb with the external fixator connected.

*Step 4* manually test bone stability after frame destabilization by removing the connecting rods. The stability was checked by gentle trials of performing translation, angulation and rotation in the different planes while noticing the patient facial expression. If any mechanical instability was present or presence of an unproportionate pain, the rods were connected again. If the tibia was stable, then we ask the patient to perform bipedal standing, bipedal walking in sequential steps.

*Step 5* repeat SLST on the treated limb after frame destabilization.

In the case of bone transport the frame was dynamized for regenerate site and docking site each one at a time. If the patient can perform the test while the frame was dynamized at only one site (either the docking or the regenerate), then the patient was encouraged to ambulate while the frame was dynamized at this site and to come to another clinical visit to examine the other site after dynamization.

The interpretation of the test was done by two experienced surgeons in limb reconstruction who must fulfil at least two of the following criteria: fellowship-trained in limb lengthening and reconstruction, perform more than 100 limb reconstruction procedures per year and/or in practice in limb reconstruction more than 10 years. If there was a contradiction between the two surgeons, the case was referred to the senior author for final decision making whether to remove the fixator or to wait.

Interpretation of the test was also done by a junior orthopedic surgeon who was unaware of the decision of the two observers. The criteria to be considered a junior orthopedic surgeon are a medical student with a clinical elective training in orthopedic surgery or a resident who hasn’t yet complete his residency program, has no fellowship training in limb reconstruction and has no experience in limb reconstruction. The interpretation of the test was recorded by all the observers in each visit.

If the patient was not able to do these steps; the decision to remove the fixator was postponed and the patient was given another appointment in the outpatient clinic after four to six weeks. In each visit the patients were evaluated by the radiological evaluation of the plain x rays and clinical evaluation by the SLST.

If there was agreement between the two observers to remove the fixator or if the decision of the senior author in case of a contradiction between the two observers was in favors of removing the fixator, the patient was given another appointment after one or two weeks to be tested again before removal of the frame.

The level of agreement between the two experienced observers were statistically analyzed using kappa static to test for inter-observer reliability during each visit for all included patients. It is also compared with the observations from the junior observer. The intra observer reliability for the SLST test was measured for the three observers during the last two appointments.

The external fixator index and incidence of refracture were compared between (Group A) and (Group B). The treatment outcome was assessed based on both objective (clinical and radiographic evaluation) and subjective criteria (limb function and patient satisfaction) using our system of results evaluation^14^. The outcome was satisfactory or unsatisfactory based on these findings (Table 1).

**Table 1:** Evaluation of the results based on clinical and radiographic evaluation.

Parameter	Satisfactory	Unsatisfactory
Bony union	United	Non-united
Residual deformity	Less than 5°	More than 5°
Residual leg-length discrepancy	Less than 2.5 cm	More than 2.5 cm
Recurrent infection	No more infection	Bone and/or soft-tissue infection
Soft-tissue healing	No exposed bone	Soft-tissue defect remaining
Permanent joint contracture	Less than 5°	More than 5°
Persistent pain	No or mild pain	Moderate or incapacitating pain
Return to previous work	Yes	Has to change job
Patient satisfaction	Satisfied	Not satisfied

**Fig. 2:**
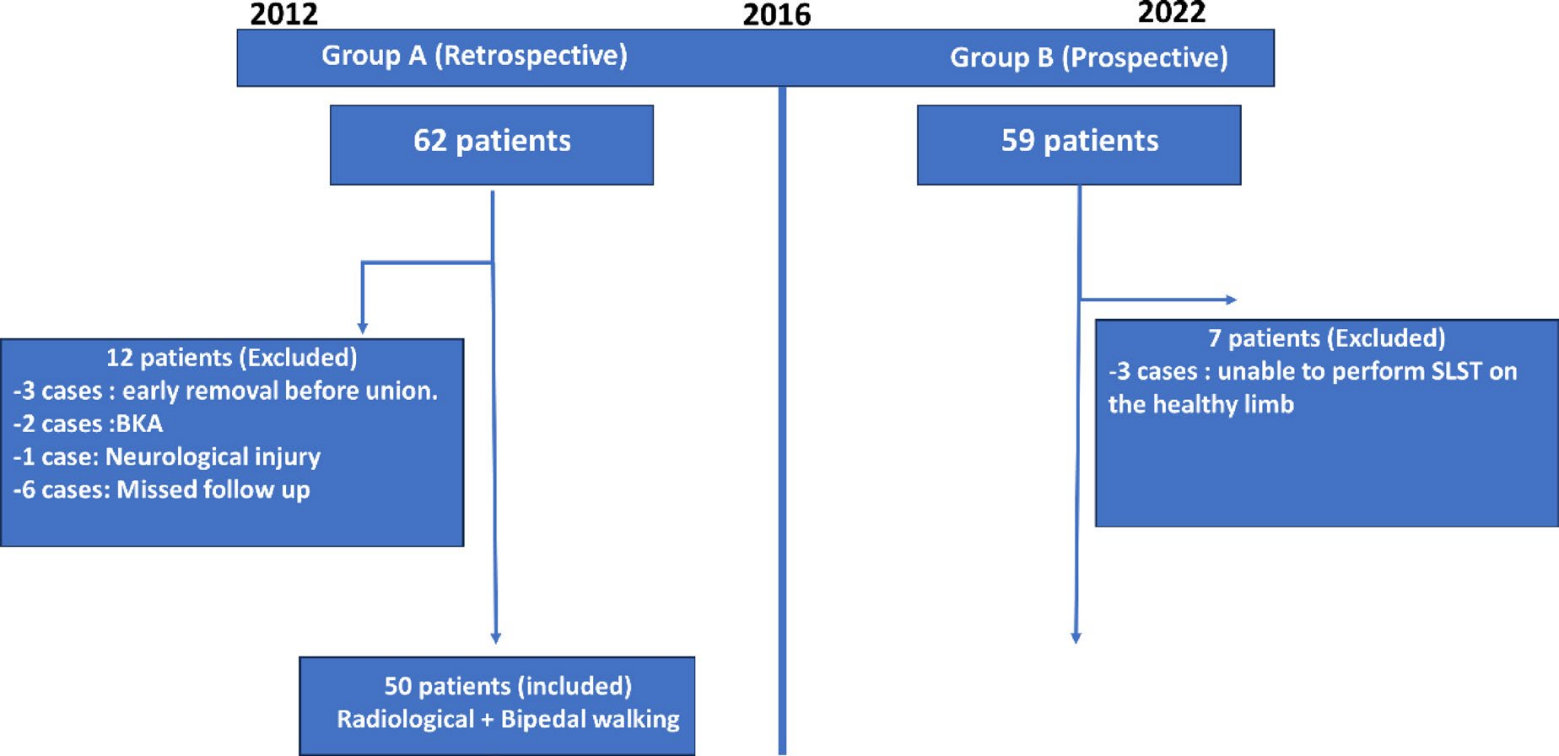
Flowchart illustrates the distribution of included and excluded cases in both groups.

From 2012 to 2015 a total of 62 patients were admitted for either tibial transport or lengthening. three patients were excluded from the study due to severe pin tract infection that necessitated early removal of the fixator before complete radiological union and application of above knee cast, below knee amputation was done in a further two cases during bone transport, one case was intolerable to the procedure and the second case due to intractable infection. One case was excluded due to presence of neurological injury in the treated limb. Further Six patients were excluded due to missing follow- up after removal of the fixator. A total of 50 patients were included in group A. (Fig. 2)

From 2016 to 2022 a total of 59 patients were admitted for tibial distraction osteogenesis. Three patients were excluded due to inability to perform the single leg stance test efficiently on the healthy limb due to being overweight, two cases did not complete the follow-up at our institution and the removal of the external fixator was done in two cases before the ability to perform the test due to severe pin tract infection. a total of 52 patients were included in group B. (Fig. 2)

## Results

The demographic data of groups A and B are summarized in (Table 2).

**Table 2:** Characteristics of group A and group B.

	Group A (radiological and bipedal walking) *n* = 50		Group B (radiological and SLST) *n* = 52	*P* value
Age	Range (20–53)		Range (18–58)	0.891
	Mean ± SD (35.7 ± 9.7)		Mean ± SD (36 ± 9.4)	
Sex	Male: *n* = 37 (74%)		Male: *n* = 34 (65.4%)	0.344
	Female: *n* = 13 (26%)		Female: *n* = 18 (34.6%)	
Site	Rt: *n* = 28 (56%)		Rt: *n* = 24 (46%)	0.320
	Lt: *n* = 22 (44%)		Lt: *n* = 28 (54%)	
Purpose of Ilizarov frame	Bone transport: *n* = 28 (56%)		Bone transport = 29 (55.8%)	0.981
	Simple leg lengthening: *n* = 22(44%)		Simple leg lengthening: *n* = 23 (44.2%)	
Co morbidities	DM	2 (4%)	3 (5.8%)	0.646
	HTN	3 (6%)	2 (3.9%)	
	HCV	1 (2%)	2 (3.9%)	
	Smoking	7 (14%)	8 (15.3%)	
External fixator index	Range (30–55)		Range (32–58)	0.018*
	Mean ± SD (40.4 ± 6.4)		Mean ± SD (43.7 ± 7.2)	
Incidence of refracture	5 cases (10%)		1 case (1.9%)	0.109
Follow up	Range (15–60) months		Range (12–60) months	0.752
	Mean ± SD (28.6 ± 11.5)		Mean ± SD (27.9 ± 11.3)	
Outcome	Satisfactory: 45 (90%)		Satisfactory:49 (94.2%)	0.426
	Unsatisfactory:5 (10%)		Unsatisfactory:3 (5.8%)	

There was no statistically significant difference in the distribution of age, sex, purpose of external fixator application and the co-morbidities between the two groups. The number of visits after agreement between the two observers about radiological union ranged from two to four visits until removal of the external fixator. The number of visits in group A was 156 visits (average 3.1 visit/patient). A total of 178 visits have been recorded from the 52 patients in Group B. (average 3.4 visit/patient)

*Inter-observer reliability* The level of agreement between the two experienced observers in the overall visits was almost perfect (kappa value 0.825). the Fleiss kappa value between the two experienced observers and the junior observer (0.882).

*Intra observer reliability* There was a substantial agreement in the intra observer reliability of the two experienced observers. Kappa value (0.658). The observations from the junior observer during the last two clinical visits for each patient were constant with the same decision.

There was a contradiction between the two observers in only 14 out of 178 visits (8%). The decision of the senior observer was not to remove the fixator and wait for another appointment in 9 out of 14 cases (64%).

On the first visit after agreement of the radiological union, there were 14 patients out of 52 patients (27%) were able to do bipedal walking however, they were not able to do SLST effectively.

The mean of the external fixator index in group A was smaller than group B which was statistically significant.

The SLST was able to predict regenerate maturation and safe removal of the external fixator in 98% of cases in our series.

**Fig. 3:**
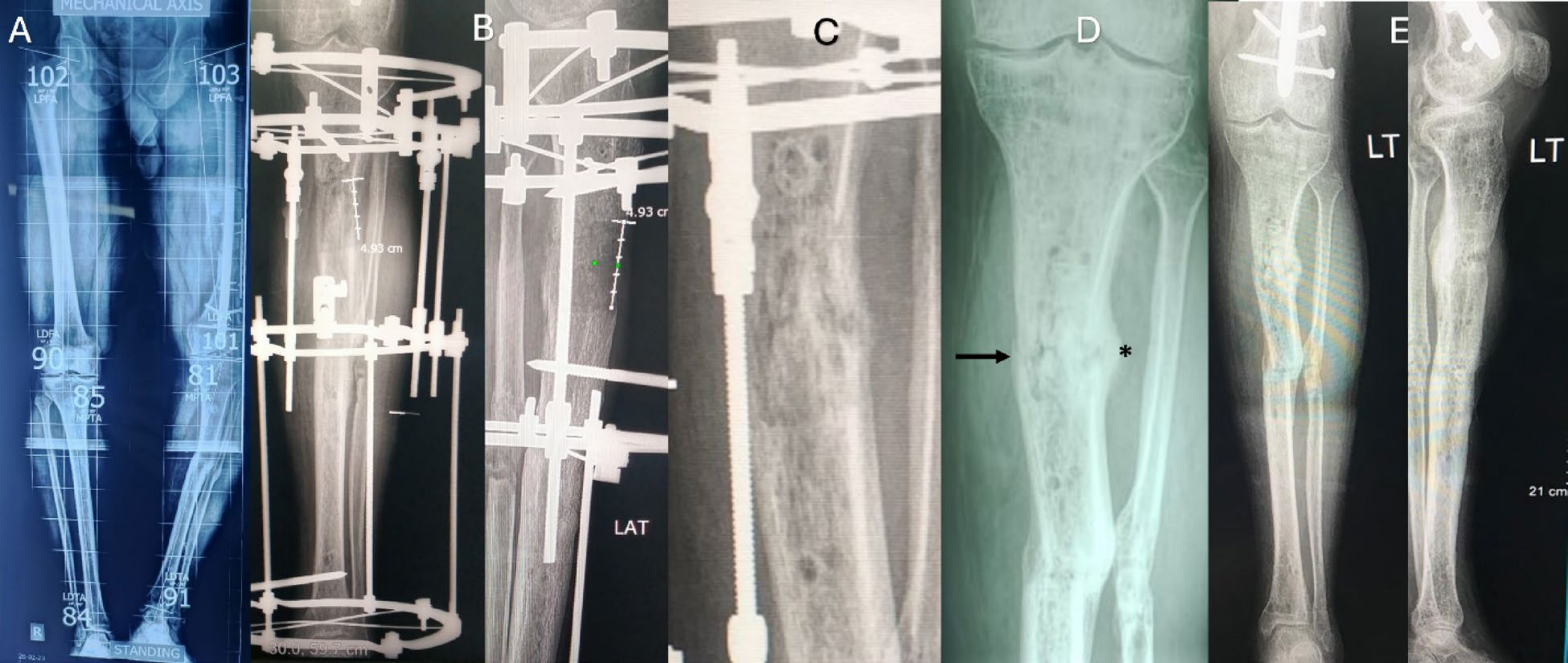
(**A**) A case of post traumatic varus deformity and Limb length discrepancy (LLD). (**B**) After correction and lengthening with Ilizarov external fixator. (**C**) Shape of the regenerate at the time of frame removal. (**D**) one month after removal of the frame, please notice the fine crack line in the middle of the regenerate (Arrow) and the newly formed callus laterally indicative of insufficiency fracture (asterisk), the patient was managed with above knee cast. (**E**) Final follow up of the patient after removal of the cast.

**Fig. 4:**
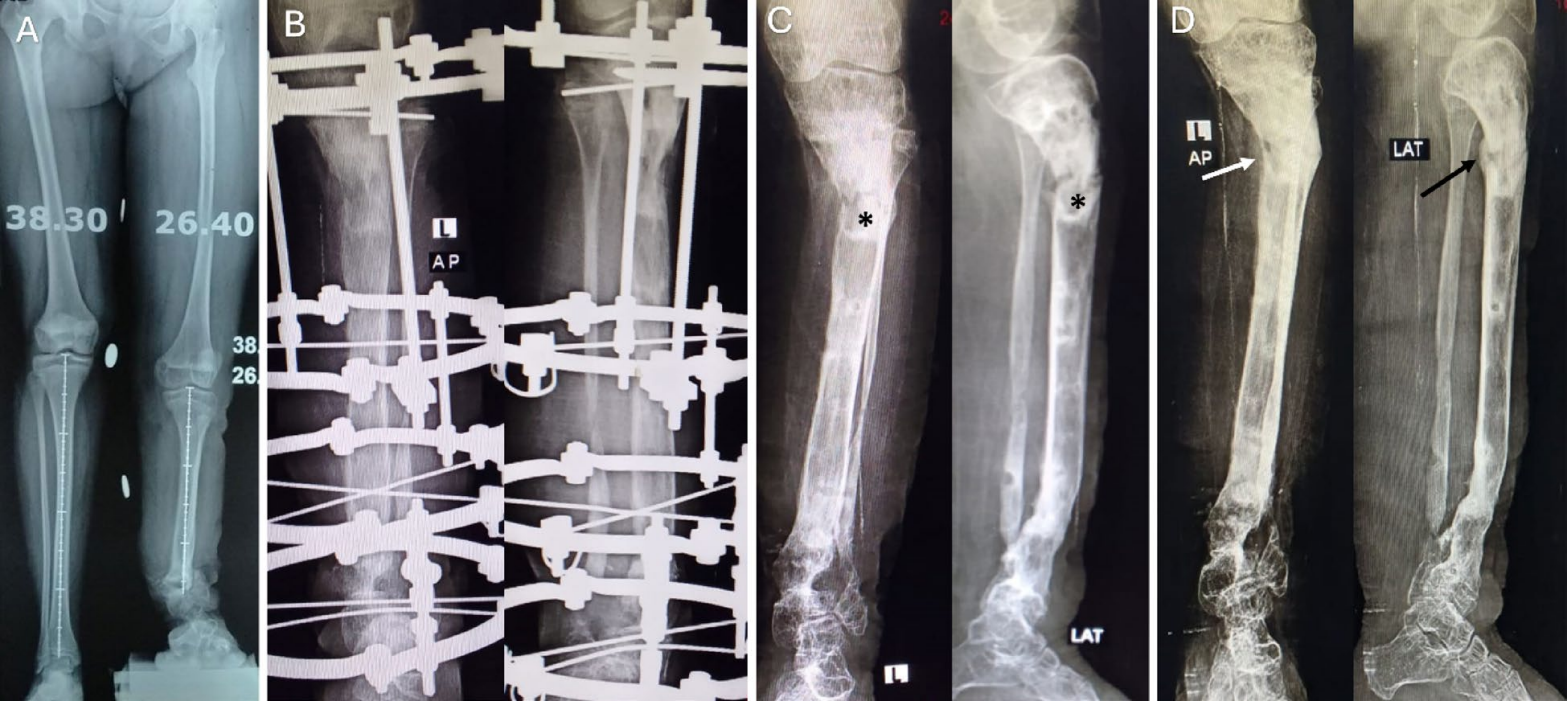
(**A**) A case of congenital stiff pseudarthrosis of the distal tibia and distal fibula with valgus deformity of the distal tibia and LLD. (**B**) Application of Ilizarov frame to correct the distal valgus deformity and lengthening of the tibia to compensate the LLD. Note the Shape of the regenerate at the time of frame removal and there was a residual valgus deformity. (**C**) Refracture and bending (asterisk) at the middle of the regenerate due to early frame removal. The patient was treated with above knee cast. (**D**) Residual deformity of the regenerate at the final follow- up after removal of the cast. Please notice the bridging callus at the posterior and medial cortex (arrows).

Refracture after external fixator removal occurred in five cases in group A (10%) compared to one case in group B (1.9%). Three cases with proximal tibia regenerate fracture, treated in above knee cast and two cases with refracture at docking site, treated with reapplication of the external fixator and iliac crest bone graft. In group B refracture was occurred in only one case with proximal regenerate fracture and treated with above knee cast. The patients with a refracture required further time ranged from 8 to 15 weeks until removal of the cast or the frame (mean 10.5 ± 2.7). the outcome was unsatisfactory in three cases in group A due to knee joint stiffness, residual deformity due to regenerate bending, and one patient was unable to return to his previous work. The cases with a refracture are summarized in (Table 3) (Figs. 3 and 4).

**Table 3:** Cases with refracture after removal of the frame.

	Group	Age	Sex	Side	Purpose of the frame	Site of the fracture	Regenerate length	Comorbidities	Further management	Time to union
Case1	A	29	Male	Rt	Bone transport	Proximal tibia (regenerate)	3 cm	–	Above knee cast	8 weeks
Case2	A	21	Male	Lt	Lengthening	Proximal tibia (regenerate)	5 cm	smoking	Above knee cast	10 weeks
Case3	A	38	Male	Lt	Bone transport	Distal tibia (Docking site)	4 cm	smoking	Reapplication of the frame and grafting	12 weeks
Case4	A	22	Female	Lt	Lengthening	Proximal tibia (regenerate)	6 cm	–	Above knee cast	15 weeks
Case5	A	39	Male	Rt	Bone transport	Proximal tibia (Docking site)	3 cm	Smoking-HTN	Reapplication of the frame and graft	10 weeks
Case6	B	22	Female	Rt	Lengthening	Proximal tibia (regenerate)	5 cm	–	Cast	8 weeks

## Discussion

Distraction osteogenesis has been used extensively to treat complex conditions using the circular external fixator. However, many complications may occur due to prolonged application of the frame^1,15^.

The decision of when to remove the external fixator is highly important. Early removal of the fixator may lead to regenerate bending or fracture. Late removal of the fixator could be complicated by pin tract infection, joint stiffness, psychological problems, and economic burden^11,16^.

There is no gold standard test to judge the maturation and stiffness of the regenerate. Most surgeons depend on combined radiological assessment of the callus and full weight bearing^11^.

We hypothesized that clinical judgement of the regenerate maturation and bone healing depending on the bipedal walking may be deceptive.

Various gait adaptation and defensive mechanisms can be recruited by the patient as a stress shielding mechanism to overcome the pain and/or weakness in bipedal walking. A decrease in walking velocity, and cadence, an increase double limb support time, a decrease in the stance phase, an increase in the swing time of the antalgic limb, and increase in the load on the healthy limb may be recruited to reduce the stresses on the antalgic limb^17^.

The single leg stance test is a simple clinical test with no need for complex devices, radiation, or high cost to judge the maturation of the regenerate, the patient couldn’t recruit the other limb to overcome the pain or deceive the observer, and its observation doesn’t depend on the bipedal gait kinematics.

Refracture after removal of the external fixator is very challenging to the patient and the treating surgeon. The reported incidence of regenerate refracture ranges from 3 to 50%^18–20^.

In our series refracture occurred in five cases out of 50 patients (10%) in group A. The refracture occurred at the regenerate in 3 cases (6%) and at the docking site in 2 cases (4%). The incidence of refracture was significantly decreased after adding the single leg stance test to our evaluation system before removal of the fixator to only one case (2%).

In our series we noticed that, about one third of patients can do bipedal walking effectively early after confirmation of radiological union, while they still couldn’t do the SLST. We called this period the “Gray zone” in which the maturation of the regenerate is questionable, and we assumed that the removal of the fixator early in this period could be risky and better delayed. Hence, the slight increase in the mean of the external fixator index in group B may be predictable due to this delay.

The external fixator index (EFI) was significantly higher in group B, with a mean of 43.7 day/cm compared to group A, which had a mean of 40.4 days/cm. Notably, the reported EFI values in the literature vary widely. For instance, a narrative review by Aktuglu et al.^21^ involving 619 patients in 27 studies treated with Ilizarov bone transport for critical-sized tibial bone defect reported a mean EFI of 1.74 months/cm range (0.28–4.2 months/cm).Some studies such as those by Xu et al.^22^, and Azzam et al.^23^ reported EFI values of 41 and 40.3 days/cm respectively, which are more comparable to the values observed in our study.

The SLST may have uncovered the fact that external fixators were being removed too early and that the use of previous assessment modalities was perhaps inadequate.

The inter observer reliability of the SLST between the three observers was almost perfect. This indicates that even the junior orthopedic surgeons can do interpretation of the test efficiently. A contradiction between the observers occurred in only 8% of the visits. The senior observer chose to delay the removal of the fixator in 9 out of 14 cases. In these cases, the senior observer noticed that five patients were using finger support during performing the SLST. The other four patients were able to perform the test without support, however, they performed the test while clenching their toes and their facial expression indicated that they were doing the test with great difficulty.

In our series all the cases with regenerate fracture or bending were treated with above knee cast. Two cases with fracture at the docking site were treated with reapplication of the frame and grafting. Comparable to our conservative treatment, Hosny et al. treated all the tibiae (23 tibiae) with refracture after Ilizarov frame removal with above knee cast converted into below knee cast after early callus formation^24^. Simpson et al. used nail, plate, External fixator or cast in the treatment of 17 refracture after distraction osteogenesis^19^.

There are some limitations in our study, it is a single center, with a relatively small number of cases, there are multiple factors may predispose to refracture other than the early removal of the fixator. The retrospective data from group A was compared with the prospective data from Group B while the outcome of the earlier cohort was known, the increased experience of the surgical team over time may have contributed to improved outcomes in the latter group. This introduces a potential confounding factor that could influence the interpretation of the results. and the absence of quantitative measures to assess regenerate bone maturation. We further recommend implementing a multicenter study including many cases and using the SLST as a clinical evaluation in adjunctive to the radiological assessment.

In conclusion, The SLST is a simple, reproducible, and reliable clinical test that can be used to evaluate the maturation of the regenerate. The SLST gives an insight into whether it is safe to remove the external fixator or delay the decision, no radiological exposure or special devices are needed.

## Data Availability

The data is available from the corresponding author upon reasonable request.
